# Single- and multiple-locus model genome-wide association study for growth traits in Dongliao black pigs

**DOI:** 10.5713/ab.25.0126

**Published:** 2025-07-11

**Authors:** Kailing Sun, Yuan Hong, Wenyu Zhang, Jiangpeng Dong, Zuohao Wen, Zhengyu Hu, Xuhui Tan, Hao Li, Ayong Zhao, Min Huang, Tao Huang

**Affiliations:** 1 Key Laboratory of Applied Technology on Green-Eco-Healthy Animal Husbandry of Zhejiang Province, College of Animal Science and Technology, College of Veterinary Medicine, Zhejiang A&F University, Hangzhou, China; 2 College of Animal Science and Technology, Fujian Vocational College of Agriculture, Fuzhou, China

**Keywords:** Average Daily Gain, Body Weight, Genome-wide Association Study (GWAS), Multiple-locus Model, Pig, Quantitative Trait Locus

## Abstract

**Objective:**

Growth traits are one of the most important economic traits in pigs, including body weight and average daily gain. However, the available genetic markers for these traits are limited, especially concerning Chinese indigenous pigs and their hybrid breeds.

**Methods:**

To identify SNP markers and candidate genes affecting body weight and average daily gain traits, we performed a genome-wide association study (GWAS) for these traits in 358 Dongliao black pigs using three single-locus and three multiple-locus models. All pigs were genotyped using the China Chip-1 porcine SNP50K BeadChip.

**Results:**

The GWAS revealed 39 significant quantitative trait loci (QTLs) affecting body weight and average daily gain traits. Among these, 26 QTLs were significantly correlated with body weight traits. Thirteen QTLs showed significant correlations with average daily gain traits. Some candidate genes associated with body weight and average daily gain traits include *MACROD2*, *ASB13*, *ATP12A*, *ZDHHC17*, *WDR37* and *TENM4*. Of the three single-locus models examined, only the general linear model identified significant SNPs, identifying a total of 27 significant QTLs, which was the largest among the models assessed. The three multiple-locus models, multiple-locus mixed-model, FarmCPU and Bayesian-information and LD iteratively nested keyway, identified 4, 12 and 13 significant QTL loci, respectively.

**Conclusion:**

We newly identified 18 QTLs that are significantly correlated with body weight and average daily gain traits. Our results provided a foundation for biomarker breeding and enhancement of body weight and average daily gain traits in pigs.

## INTRODUCTION

Pork is the most consumed red meat in the world, and it is an important source of animal protein for humans. As of July 2024, global pork production exceeded 120 million tons. This accounts for about 35% of global meat production. Global pork consumption is expected to further increase in the future (https://www.statista.com). Growth traits are one of the most important economic traits of pigs, which are regulated by multiple genes and have complex regulation mechanisms [1]. Understanding the effect of genetic improvement on growth performance in pigs is one of the most important topics in the fields of animal genetics and breeding.

With the rapid advancements in high-density single nucleotide polymorphism arrays, genome-wide association studies (GWAS) have become a common method to study the genetic mechanism of economic traits in livestock [2–4]. Among the growth traits in pigs, body weight (BW) and average daily gain (ADG) stand as significant parameters. As of August 2024, a total of 3,698 quantitative trait loci (QTLs) were found to be associated with growth traits in the pig QTL database [5]. Among these, 1,381 QTLs specifically relate to ADG, highlighting the genetic complexity in this important growth parameter of pigs. Many previous studies have used GWAS to discover QTLs and genes associated with growth traits. For example, a pleiotropic QTL was found on *Sus scrofa* chromosome (SSC) 1 with a haplotype block of 2.19 Mb that affected the ADG in Duroc pig populations [6]. GWAS and meta-analyses of three Danish pig breeds and within-breed subpopulations identified 15 QTLs associated with ADG [7]. GWAS in offspring from Duroc × Erhualian hybridization identified *NDUFAF6*, *TNS1*, and *HMGA1* as candidate genes linked to BW and ADG [8]. GWAS in a White Duroc×Erhualian F_2_ intercross and a Chinese Sutai half-sib population identified *HMGA1* and *PLAG1* as key candidate genes for BW and ADG [9]. The candidate genes *PHLPP1*, *STC1*, *DYRK1B*, and *PIK3C2A* were detected through GWAS analysis of ADG traits in 3770 American Duroc pigs and 2090 Canadian Duroc pigs [6].

Mixed linear model (MLM) is the most commonly used model in genome-wide association analysis and is widely used to identify important loci for complex traits in animals, plants, and humans [10]. The MLM-based single-locus GWAS is proved to be feasible. However, the statistical power of GWAS based on MLM is limited for low heritability and small effect traits [11]. In order to detect more quantitative trait nucleotides (QTNs) with a low false positive rate, GWAS based multiple-locus methods were developed. The multiple-locus method of fixed and random model Circulating Probability Unification (FarmCPU) improves statistical power compared to MLM [12]. In addition, there are many other multiple-locus methods that can perform GWAS on complex quantitative traits, such as multiple-locus mixed-model (MLMM) [13] and Bayesian-information and LD iteratively nested keyway (BLINK) [14].

The growth traits are typical quantitative traits and are controlled by polygenetic mutations, each of which has a small effect, acting additively across the genome [1]. We used both single-locus and multiple-locus models GWAS on the growth traits of Dongliao black pigs in order to discover more QTLs while controlling the false positive rate. The Dongliao black pig is a breed that has been developed by selective breeding between the Chinese indigenous Min pig and the Berkshire pig [15]. In this study, the BW of 358 Dongliao black pigs at four different ages was recorded and the GWAS for both BW and daily gain was performed. The significant SNPs and candidate genes obtained by GWAS can be used as markers for genetic improvement of pig growth traits.

## MATERIALS AND METHODS

### Experimental animals and phenotype

All 358 Dongliao black pigs used in this study were raised in TianSheng. These pigs were managed and reared under the same environmental conditions. Ear tissue samples were collected from each individual, preserved in 75% alcohol and stored at −20°C. BW phenotypes were recorded at four time points, including birth weight (BW0), BW at the ages of 26 (BW26), 60 (BW60) and 90 (BW90) days. ADGs were calculated between pairs of the four time points.

### Genotyping and quality control

Genomic DNA from all pigs was extracted from ear tissue using a standard phenol/chloroform method. The concentration and quality of the DNA was measured using a Nanodrop 100 spectrophotometer (Thermo Fisher Scientific) and agarose gel electrophoresis. All DNA samples were genotyped using the China Chip-1 porcine SNP50K BeadChip according to the manufacturer’s protocol. A total of 57,466 SNPs were obtained. The quality control of the data was performed using PLINK v1.90 with the following parameters: an SNP call rate >90%, a minor allele frequency >0.01, an individual genotype missing rate <10% and Hardy–Weinberg equilibrium test p>10^−6^ [16]. After quality control, a total of 50,739 SNPs from 358 individuals were retained for further analyses.

### Genome-wide association analysis

The kinship matrix was constructed using PLINK v1.90 software [16]. GWAS were performed for the growth traits using GAPIT (version 3.0) [17]. The GWAS used three single-locus models of general linear model (GLM) [18], MLM [10], settlement of MLMs under progressively exclusive relationship (SUPER) [19], and three multiple-locus models of MLMM [13], FarmCPU [12] and BLINK [14], respectively.

The GLM model is described as follows:


(1) Y=Xβ+e

Where *y* represents the vector of observed phenotypes; *X* is the known design matrix containing the fixed effects (e.g., genetic markers, population structure factors); *β* is the unknown vector of coefficients corresponding to the fixed effects, and *e* is the unobserved vector of residuals.

The MLM model is described as follows:


(2) y=Xβ+Zu+e

Where the components *y*, *X*, *β* and *e* have the same interpretations as in GLM model. While *Z* is a known design matrix corresponding to the random effects, and *u* is the unknown vector of random additive genetic effects arising from multiple background quantitative trait locis or other sources of individual-specific variability.

The SUPER model uses the efficient algorithm and solving Eq. 2 involves determining all the unknown parameters under which the observations (*y*) have the maximum likelihood, defined as the following:


(3) $$L(y|\beta ,\;\sigma_{a}^{2},\;\sigma_{e}^{2})$$

To perform a GWAS, marker effect (*v*) is added to Eq. 2, one at a time:


(4) $$y=W_{\nu }+X\beta +e$$

Where *W* is the incidence matrix for *v*.

The MLMM model involves a forward-backward stepwise linear mixed-model regression for analyzing SNPs.

The FarmCPU model is described as follows:


(5) $$y_{i}=S_{i1}b_{1}+S_{i2}b_{2}+\ldots +S_{it}b_{t}+H_{ij}d_{j}+e_{i}$$

Where *y_i_* is the vector of phenotypic values for the *i*th pigs; *S_i_*_1_, *S_i_*_2_,_._ *…, S_it_* are the genotypes of tthe pseudo QTNs; *b_1_*,*b_2_*, *…*,*b_t_* are the corresponding effects of the pseudo QTNs; *H_ij_* is the *j*th genotype of the *i*th pig; *d_j_* is the corresponding effect of the genotype; and *e_i_* is the residuals of the model.

The BLINK model is described as follows:


(6) $$y_{i}=S_{i1}b_{1}+S_{i2}b_{2}+\ldots +S_{it}b_{t}+S_{ij}d_{j}+e_{i}$$

(7) $$y_{i}=S_{i1}b_{1}+S_{i2}b_{2}+\ldots +S_{it}b_{t}+e_{i}$$

(8) BIC=-2LL+2tLn(n)

Where *LL* is the log likelihood, *t* is the number of pseudo QTNs, *Ln* is the natural log, *n* is the number of individuals, and the symbolic meanings of Eqs. 6, 7 are the same as those of Eq. 6 in FarmCPU model.

Genome-wide significance thresholds were set as 0.05/N (0.05/50739) with Bonferroni adjustment, and the suggestive significance threshold was set to be 1/N (1/50739), where N is the number of filtered SNPs in the data set. The Manhattan plot were drawn with the CMplot package [20] and ggplot in the R software (version 4.2.2).

### Linkage disequilibrium analysis

The linkage disequilibrium (LD) blocks were identified in the chromosomal regions containing the identified significantly associated SNPs using the software Haploview v4.2 (28). The LD blocks were defined using Haploview based on default parameters according to the criteria.

### SNP annotation

The candidate genes containing or near the significant SNPs were annotated using Ensembl annotation of the *Sus scrofa* reference genome (version 11.1) ( http://www.ensembl.org/Sus_scrofa/Info/Index). The potential functions of the candidate genes were investigated by searching NCBI (https://www.ncbi.nlm.nih.gov/) and Genecards (*https://www.genecards.org*).

## RESULTS

### The descriptive statistics of the growth traits

**Figure 1:**
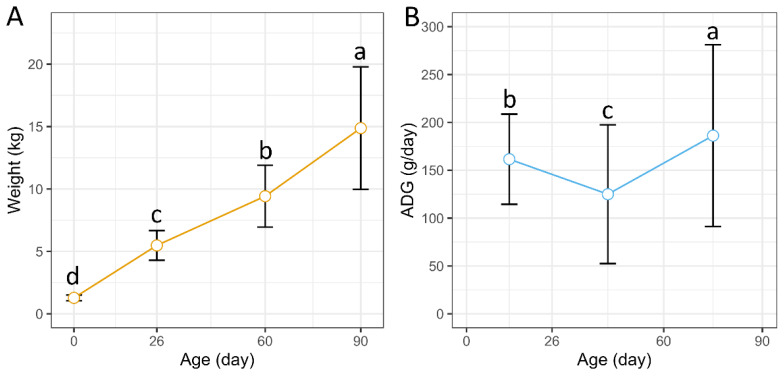
Phenotypic of growth trait. (A) Body weight at different ages. (B) The average daily gain at different time intervals. Significant differences in pairwise comparisons across age groups were analyzed using the Wilcoxon rank sum test. ^a–d^ Different superscript letters in the bar indicate significant differences (p<0.05).

We recorded the BW of 358 Dongliao black pigs at 4 time points and the ADG phenotype at 6 time intervals. The descriptive statistics for BW and ADG are presented in Figure 1 and Supplement 1. There was a significant increase in BW over age (Kruskal-Wallis test, p = 4.43×10^−189^). The BW for days 0, 26, 60, and 90 were 1.28±0.23 kg, 5.48±1.19 kg, 9.42±2.48 kg, 14.88±4.91 kg, respectively (Figure 1A, Supplement 1). The coefficients of variation (CV) of BW ranging from 17.79% to 32.98%. The ADG from 0 to 26 days was 161.58±47.08 g/day, while the ADG from 26 to 60 days dropped to 124.98±72.5 g/day, but the ADG from 60 to 90 days significantly increased to 186.19±94.96 g/day (Wilcoxon rank-sum test, p<0.05) (Figure 1B and Supplement 1).

### Genome-wide association analysis of body weight traits

**Figure 2:**
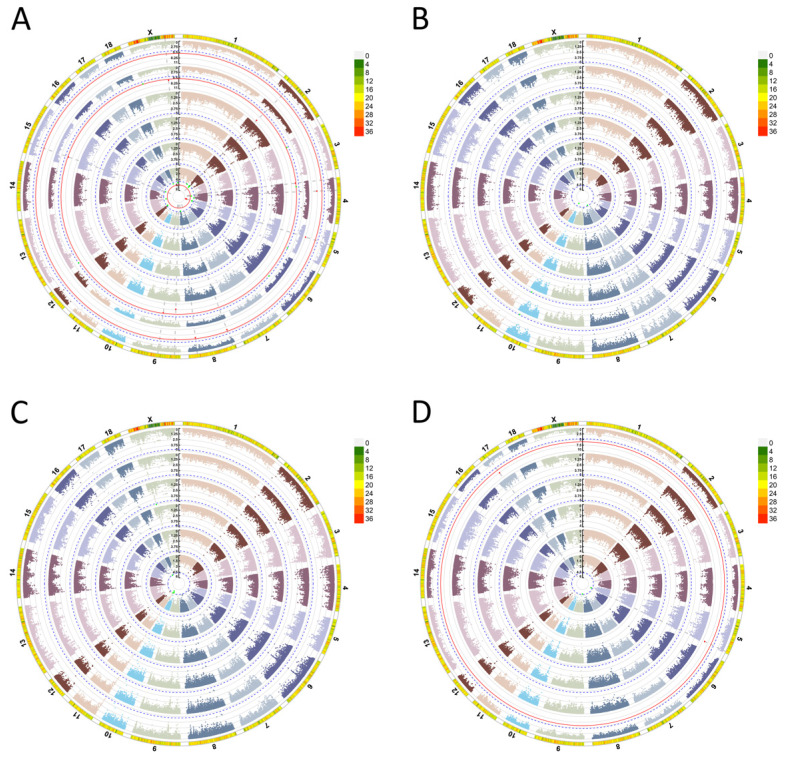
Genome-wide association study (GWAS) analysis of body weight (BW) traits. (A) BW0. (B) BW26. (C) BW60. (D) BW90. The Manhattan plot are GLM, MLM, SUPER, MLMM, FarmCPU and BLINK models from the inside to the outside. The outermost lines are the density distribution of detected single nucleotide polymorphisms (SNPs). A more reddish hue in the outermost sector signifies a higher density of SNPs in that chromosomal region, whereas a grayer tone indicates lower SNP density. GLM, general linear model; MLM, mixed linear model; SUPER, settlement of MLMs under progressively exclusive relationship; MLMM, multiple-locus mixed-model; BLINK, Bayesian-information and LD iteratively nested keyway.

To find the potential loci associated with BW traits, single- and multiple-locus model GWAS were performed using the filtered 50,739 SNPs in 358 individuals. For the BW0 trait, 10, 9, and 3 significant QTLs were detected by the GLM, FarmCPU, and BLINK models, respectively (Figure 2A and Table 1). The QTLs on SSC 2, 4, 5, 15 and X were detected by at least two models. A total of 8 SNPs were detected on SSC 2 that exceeded the significance threshold (p<1/50739). Among them, the CNC10020767 SNP was close to the upstream *FANCF* gene. The top SNP CNCB10003210 was located at 39,755,227 bp of SSC 4 with p value of 3.35×10^−9^ (BLINK) and 2.09×10^−6^ (GLM). The effect value was −0.11 kg (A/G). Notably, the top SNP was found to be located within the *CPQ* gene. On SSC 5, both GLM and BLINK models detected SNPs associated with BW0. These SNPs exceeded the genome-wide significance threshold (p<0.05/50739). The most significant SNP located at 41,950,115 bp (CNC10050778) with an effect value of more than 0.2 kg (A/G). Significant QTLs have been identified on SSC15 and SSCX, which are located within the *MYOM2* gene and upstream of the *F9* gene, respectively.

**Figure 3:**
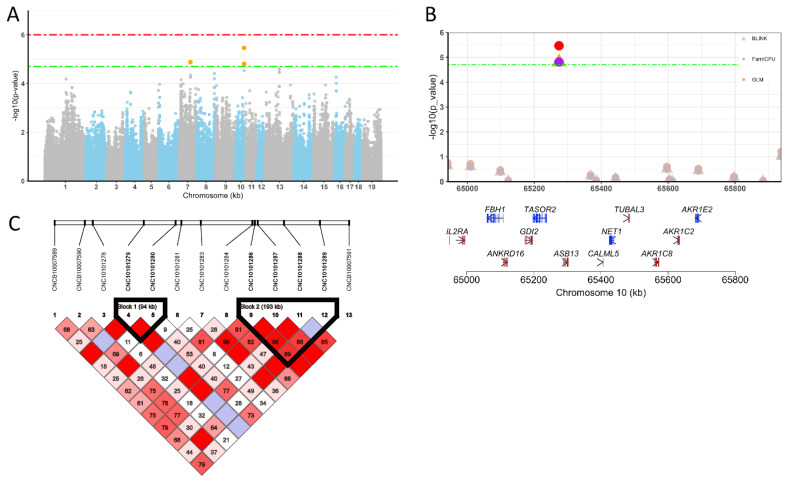
Genome-wide association study (GWAS) analysis of BW26. (A) Manhattan plot of the GWAS for BW26 trait by GLM model. The green dashed line represents chromosome-wide significance threshold. The red dashed line represents genome-wide significance threshold. The orange dots indicate that the p-value of the SNPs exceeds the chromosome-wide significance threshold. (B) Regional GWAS results on chromosome 10 for BW26 using GLM, FarmCPU and BLINK models. The green dashed line denotes the chromosome-wide significance threshold. Red dots represent SNPs identified by the GLM model, purple dots indicate loci detected by the FarmCPU model, and orange triangles signify loci identified by the BLINK model. (C) Haplotype block of a significant region (800 kb) of SSC10. BW, body weight; GLM, general linear model; BLINK, Bayesian-information and LD iteratively nested keyway.

For the BW26 trait, SNPs on SSC 7 and 10 were found to exceed the significance threshold (p<1/50739) (Table 1, Figures 2B, 3A). Among them, only one SNP was detected on SSC 7 in the GLM model GWAS that exceeded the suggestive significance threshold (p<1/50739). On SSC 10, the GLM, FarmCPU and BLINK models GWAS detected SNPs associated with BW26. These SNPs exceeded the suggestive significance threshold (p<1/50739). The most significant SNP located at 65,273,844 bp (CNC10101279) with an effect size of −0.97 kg (A/G). The most significant SNP was located at 1,688 bp upstream of the *ASB13* gene (Figure 3B and Table 1). The most significant SNP was located in an LD block of length 94 kb (Figure 3C).

GWAS for BW60 traits found that GLM and MLMM models detected 5 QTLs on SSC 10, 11, 17 and X that exceeded the significance threshold (p<1/50739). However, no SNPs were detected by both models (Table 1 and Figure 2C). For the BW90 trait, SNPs on SSC 5 and 17 were found to exceed the genome-wide significance threshold (p<0.05/50739) by the BLINK model GWAS. In addition, the GLM model GWAS for the BW90 trait found that the SNPs on SSC 5 and 9 exceeded the suggestive significance threshold (p<1/50739). The SNP of CNC10052030 at 106,863,651 bp of SSC 5 was detected by both BLINK and GLM model GWAS, with an effect value of −3.54 kg (A/G). The nearest gene to CNC10052030 was the *ZDHHC17* gene located at 2,469,391 bp upstream (Table 1 and Figure 2D). The same significant QTL was found on SSC 17 for BW60 and BW90 traits GWAS. The most significant SNP of the QTL was located at 23,852,682 bp of SSC 17, within the *MACROD2* gene.

### Genome-wide association analysis of average daily gain traits

**Figure 4:**
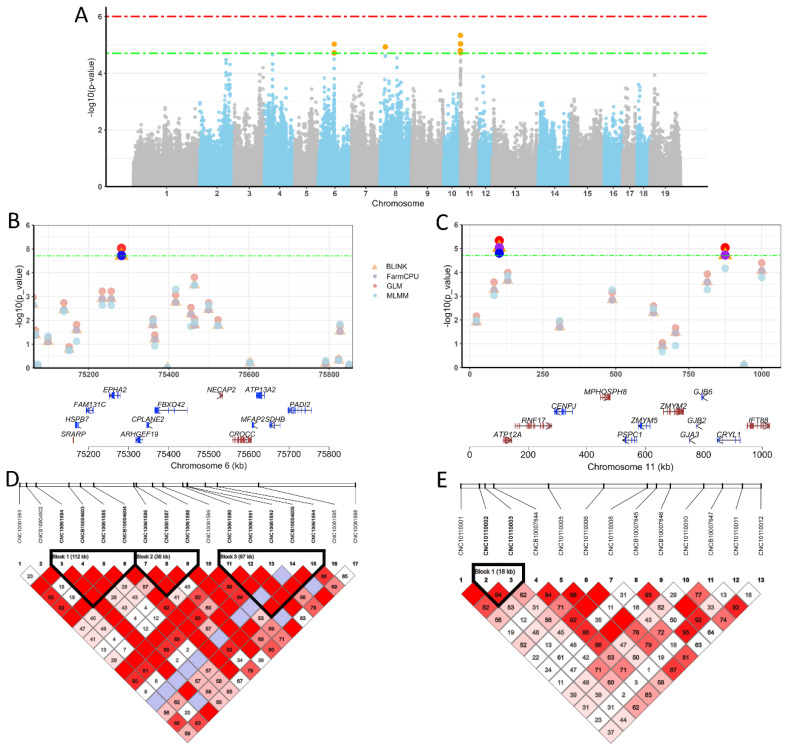
Genome-wide association study (GWAS) analysis of ADG26-60. (A) Manhattan plot of the GWAS for BW26-60 trait by GLM model. The green dashed line represents chromosome-wide significance threshold. The red dashed line represents genome-wide significance threshold. The orange dots indicate that the p value of the SNPs exceeds the chromosome-wide significance threshold. (B) Regional GWAS results on SSC 6 for BW26-60 using GLM, MLMM, FarmCPU and BLINK models. (C) Regional GWAS results on chromosome 11 for BW26-60 using GLM, MLMM, FarmCPU and BLINK models. The green dashed line denotes the chromosome-wide significance threshold. Blue dots represent SNPs identified by the MLMM model, red dots denote SNPs detected by the GLM model, purple dots indicate loci identified by the FarmCPU model, and orange triangles signify loci detected by the BLINK model. (D) Haplotype block of a significant region (800 kb) of SSC6. (E) Haplotype block of a significant region (800 kb) of SSC11. ADG, average daily gain; BW, body weight; GLM, general linear model; MLMM, multiple-locus mixed-model; BLINK, Bayesian-information and LD iteratively nested keyway.

For ADG traits, we also used three single-locus and three multiple-locus models to perform GWAS. We detected 5, 3, 3 and 2 significant QTLs for ADG26-60, ADG0-60, ADG0-90 and ADG26-90 traits, respectively (Table 1 and Supplement 2). For the ADG0-26 and ADG60-90 traits, we found no SNPs above the significance threshold (p<1/50739). Four models identified SNPs exceeding the significance threshold on SSC 6, 8, and 11 for the ADG26-60 trait (Table 1 and Supplement 2). On SSC 6, the SNP at 75,282,198 bp was found to exceed the suggestive significance threshold (p<1/50739) detected by the BLINK, FarmCPU, GLM and MLMM models. The nearest gene to the SNP (CNCB10004604) was the *EPHA2* gene located at 2,884 upstream (Figure 4B). The most significant SNP (CNCB10004604) was located in an LD block of length 112 kb. The block contains 4 SNP and the r^2^ = 1.0 (Figure 4D). On SSC 11, we detected a total of 7 SNPs reaching the suggestive significance threshold (p<1/50739), with the most significant SNP (CNC10110003) located at 100,808 bp. The *ATP12A* gene is the closest to the SNP (CNC10110003), located 13,517 bp downstream of the SNP (Figure 4C). The most significant SNP (CNC10110003) was located in an LD block of length 18 kb (Figure 4E). For ADG0-60 trait, 5 QTLS were detected by GLM and MLMM model GWAS, but any QTL was detected by only one model (Table 1 and Supplement 2). For the ADG0-90 trait, SNPs on SSC 1, 5 and 17 were found to exceed the significance threshold (p<1/50739). Among them, only the SNP located at 106,863,651 bp on SSC 5 was detected by both BLINK and GLM model GWAS. The most significant SNP has an effect size of −46.35 g/day, and its nearest gene was the upstream 2,469,391 bp *ZDHHC17* gene (Table 1 and Supplement 2). For the ADG26-90 trait, SNPs on SSC 5 and 9 were found to exceed the significance threshold (p<1/50739) detected by the BLINK and GLM models (Table 1 and Supplement 2). The most significant SNP of CNC10052030 at 106,863,651 bp of SSC 5 has an effect value of more than −41 g/day (A/G), and is located at 2,469,391 bp downstream of the *ZDHHC17* gene. On SSC 9, the most significant SNP was located at 13,348,686 bp with an effect value of −99.84 g/day (GLM model, A/G), and the SNP was located within the *TENM4* gene (Table 1).

### Comparison of six models for detection of significant quantitative trait loci

**Figure 5:**
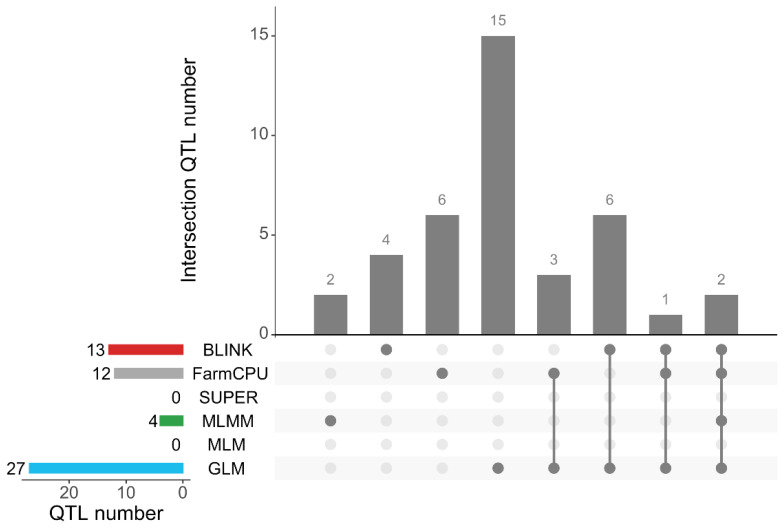
The upset plot shows the number of QTLs detected by the GLM, MLM, SUPER, MLMM, Farm-CPU and BLINK models. The left bar chart shows the total QTL count for each method. Grey bars represent the number of shared QTLs in each category, while dots indicate that QTLs have been detected by the corresponding method. QTLs, quantitative trait loci; BLINK, Bayesian-information and LD iteratively nested keyway; SUPER, settlement of MLMs under progressively exclusive relationship; MLM, mixed linear model; GLM, general linear model; MLMM, multiple-locus mixed-model.

We used 6 different models to perform GWAS analysis on BW and ADG traits and detected a total of 39 QTLs (Figure 5 and Table 1). Among the three single-locus models GLM, MLM and SUPER, only the GLM model detected 27 significant QTLs, while neither the MLM nor the SUPER models detected QTL loci that reached the suggestive significance threshold (p<1/50739). The three multiple-locus models, MLMM, FarmCPU and BLINK, identified 4, 12 and 13 significant QTL loci, respectively. Among these significant QTL loci detected, two were detected by four models: GLM, MLMM, FarmCPU and BLINK. Only one significant QTL locus was detected by the three models (GLM, FarmCPU and BLINK). There were 9 significant QTL loci detected by two models at the same time. There were 27 significant QTL loci detected by only one model, among which 15, 2, 6 and 4 significant QTL loci were detected by GLM, MLMM, FarmCPU and BLINK models, respectively.

## DISCUSSION

In this study, we performed GWAS on 358 pigs using three single-locus and three multiple-locus models and identified 39 significant QTLs associated with BW and ADG traits. Among them, 18 QTLs were newly discovered compared to the pig QTL database. BW and ADG are important indicators of breeding performance at different stages of development, from birth right through to adulthood. BW and ADG are classical quantitative variables with moderate to high heritability, which lends significant support to the polygenic inheritance paradigm [21–23]. In addition, the strong genetic correlation between the BW and ADG traits and the identification of 6 QTLs common to both traits suggest that these traits may share similar genetic components or be influenced by certain pleiotropic genomic regions [1].

An identical QTL on SSC 17 was found to be significantly associated with BW60, BW90, ADG0-60 and ADG0-90 traits in this study. The most significant SNP in this QTL was located in *MACROD2* gene. The *MACROD2* gene encodes the mono-ADP-ribosyltransferase two, which catalyses ADP-ribosylation [24]. It was found that *MACROD2* gene affected the backfat thickness of pigs by affecting fat metabolism [25]. Some studies have found that the *MACROD2* gene is associated with net meat weight in beef cattle [26]. Meanwhile, human studies have shown that deletion of the *MACROD2* gene exon was closely associated with obesity [27]. The *MACROD2* gene has been reported to modulate adipogenesis [28]. By affecting fat metabolism, the MACROD2 gene may affect the BW and daily weight gain of Dongliao black pigs. Previous studies of femoral microCT screening in knockout mice versus normal controls found that *MACROD2* was a novel candidate gene for bone regulation [29]. It was found that homozygous *MACROD2* knockout mice had increased bone mineral density, decreased body length and lean mass [30]. In addition, another study found that the *MACROD2* gene was significantly associated with bone density [31]. Therefore, *MACROD2* gene may affect both BW and daily gain of Dongliao black pigs by influencing bone development.

Genome-wide association analysis of GLM, FarmCPU and BLIINK models in BW26 trait found a significant QTL on SSC10, in which *ASB13* gene was a possible candidate gene. The *ASB13* is a member of the ankyrin repeat and suppressor of cytokine signal (SOCS) box E3 ligase protein family [32]. Genetic polymorphisms in *ASB13* have been found to be associated with an increase in the size of the fat compartment and morbid obesity [33,34]. *ASB13* gene may affect the BW of Dongliao black pigs by influencing fat formation. For the BW60, we found that *ATP12A* gene was a possible candidate gene. The *ATP12A* gene encodes the catalytic subunit of the non-gastric proton pump, which belongs to the group of X/K-ATPases of the P2-type ATPase family and mediates the secretion of protons in exchange for potassium ions [35,36]. The ATP12A protein exhibits a broad expression pattern across mammalian tissues [37,38]. The *ATP12A* gene is localized to the apical membrane of normal mouse colonic epithelial cells and may influence the pH of the gut environment by regulating proton transport [38]. Then modulate gut microbiota can improve growth performance of nursery pigs [39].

## CONCLUSION

Among the six models used in this study, BLINK, FarmCPU, and GLM performed well in identifying significant SNP markers for BW and ADG, while MLM model failed to detect significant SNPs for these traits. The results from the efficient models (GLM, BLINK, FarmCPU) were consistent with findings from studies on cattle [40], sheep [41], and pig [1]. However, this study also used the SUPER model, which failed to identify QTLs for the BW and ADG traits, and the MLMM model, which showed moderate performance. This could be due to the fact that MLM and SUPER models are appropriate for scenarios with significant population structure (for example, geographic diversity or family relatedness). However, all pig of this study were all from the same region, and the GAPIT software automatically generated a kinship. This may have led to model overfitting and resulted in false-negative findings. The BLINK model performed better than the FarmCPU model, which is consistent with previous studies [14].

## Figures and Tables

**Table 1:** The significant loci associated with BW and ADG traits

Trait	Model	Chr	Position	Top SNP	N_genome_^1)^	N_suggest_^2)^	p-value	Alleles	Effect	Nearest gene^3)^	Distance (bp)^4)^
BW0	BLINK	4	39755227	CNCB10003210	1	1	3.35E-09	A/G	−0.11	CPQ	0
BW0	BLINK	5	41950115	CNC10050778	1	1	1.10E-11	A/G	0.20	BICD1	0
BW0	BLINK	8	9152556	CNC10080168	2	2	1.62E-07	A/G	0.08	NKX3-2	−26,676
BW0	FarmCPU	2	36919501	CNC10020767	1	1	4.50E-11	A/G	−0.08	FANCF	33,645
BW0	FarmCPU	3	19429439	CNC10030392	0	1	2.80E-06	A/G	0.06	GTF3C1	0
BW0	FarmCPU	4	2990838	CNC10040074	1	2	6.15E-07	A/G	−0.05	AGO2	0
BW0	FarmCPU	5	107066508	CNC10052032	0	1	1.51E-05	A/G	0.04	ZDHHC17	2,672,248
BW0	FarmCPU	6	107560985	CNCB10004764	0	1	1.45E-05	A/G	0.07	GATA6	246,359
BW0	FarmCPU	9	18195412	CNC10090401	2	2	3.15E-09	C/A	0.07	DLG2	0
BW0	FarmCPU	13	1796416	CNCB10008343	0	1	1.41E-05	A/G	−0.04	PIK3R4	0
BW0	FarmCPU	15	33549017	CNC10150672	1	1	5.96E-07	A/G	0.08	MYOM2	0
BW0	FarmCPU	X	114122328	CNC10233248	1	2	2.82E-07	A/G	−0.10	F9	−96,379
BW0	GLM	1	277105135	CNC10014922	0	1	1.49E-05	A/G	−0.22	OLFM1	2,843,807
BW0	GLM	2	36919501	CNC10020767	0	6	4.61E-06	A/G	−0.09	FANCF	33,645
BW0	GLM	4	39755227	CNCB10003210	0	3	2.09E-06	A/G	−0.11	CPQ	0
BW0	GLM	5	41950115	CNC10050778	2	4	2.43E-08	A/G	0.21	BICD1	0
BW0	GLM	8	122574461	CNC10082465	0	1	1.32E-05	C/A	0.08	STPG2	132,877
BW0	GLM	12	53376936	CNCB10008287	0	2	8.25E-06	C/A	−0.09	VAMP2	−1,473
BW0	GLM	13	200912860	CNCB10009480	0	1	2.95E-06	A/G	0.09	VPS26C	0
BW0	GLM	15	33549017	CNC10150672	0	1	7.88E-06	A/G	0.12	MYOM2	0
BW0	GLM	18	40720548	CNC10180766	0	1	6.48E-06	A/G	−0.08	AVL9	0
BW0	GLM	X	114122328	CNC10233248	0	1	7.32E-06	A/G	−0.13	F9	−96,379
BW26	BLINK	10	65273844	CNC10101279	0	1	1.54E-05	A/G	−0.97	ASB13	−1,688
BW26	FarmCPU	10	65273844	CNC10101279	0	1	1.54E-05	A/G	−0.97	ASB13	−1,688
BW26	GLM	7	87847151	CNC10071802	0	1	1.32E-05	A/G	−0.47	SV2B	80816
BW26	GLM	10	65273844	CNC10101279	0	1	3.42E-06	A/G	−0.97	ASB13	−1,688
BW60	GLM	10	68483334	CNCB10007604	0	1	1.31E-05	A/G	1.78	WDR37	0
BW60	GLM	11	100808	CNC10110003	0	2	5.48E-06	A/G	−1.25	ATP12A	−13,517
BW60	GLM	17	23852682	CNC10170422	0	1	1.62E-05	A/G	2.08	MACROD2	0
BW60	MLMM	X	22001250	CNC10230760	0	1	1.13E-05	C/A	1.28	MAGEB18	−81,762
BW90	BLINK	5	106863651	CNC10052030	1	1	8.06E-10	A/G	−3.54	ZDHHC17	2,469,391
BW90	BLINK	17	23852682	CNC10170422	1	1	2.91E-08	A/G	4.95	MACROD2	0
BW90	GLM	5	106863651	CNC10052030	0	1	9.53E-06	A/G	−3.71	ZDHHC17	2,469,391
BW90	GLM	9	13348686	CNC10090293	0	1	1.41E-05	A/G	−6.36	TENM4	0
ADG0-60	GLM	8	25256362	CNC10080474	0	1	1.82E-05	A/G	−26.67	PCDH7	1,383,355
ADG0-60	GLM	10	68483334	CNCB10007604	0	1	8.28E-06	A/G	30.6	WDR37	0
ADG0-60	GLM	11	100808	CNC10110003	0	2	6.66E-06	A/G	−20.71	ATP12A	−13,517
ADG0-60	GLM	17	23852682	CNC10170422	0	1	4.62E-06	A/G	37.22	MACROD2	0
ADG0-60	MLMM	X	22001250	CNC10230760	0	1	1.49E-05	C/A	21.27	MAGEB18	−81,762
ADG0-90	BLINK	1	42951497	CNC10010889	0	1	9.80E-06	A/G	26.42	FAM184A	−51,893
ADG0-90	BLINK	5	106863651	CNC10052030	1	1	7.39E-12	A/G	−46.35	ZDHHC17	2,469,391
ADG0-90	BLINK	17	23852682	CNC10170422	0	1	1.24E-06	A/G	45.11	MACROD2	0
ADG0-90	GLM	5	106863651	CNC10052030	0	1	1.21E-05	A/G	−41.06	ZDHHC17	2,469,391
ADG26-60	BLINK	6	75282198	CNCB10004604	0	1	1.93E-05	A/G	−47.96	EPHA2	2,884
ADG26-60	BLINK	11	100808	CNC10110003	0	2	9.15E-06	A/G	−40.04	ATP12A	−13,517
ADG26-60	FarmCPU	6	75282198	CNCB10004604	0	1	1.93E-05	A/G	−47.96	EPHA2	2,884
ADG26-60	FarmCPU	11	100808	CNC10110003	0	2	9.15E-06	A/G	−40.04	ATP12A	−13,517
ADG26-60	GLM	6	75282198	CNCB10004604	0	1	9.34E-06	A/G	−47.96	EPHA2	2,884
ADG26-60	GLM	8	25256362	CNC10080474	0	1	1.17E-05	A/G	−51.83	PCDH7	1,383,355
ADG26-60	GLM	11	100808	CNC10110003	0	2	4.61E-06	A/G	−40.04	ATP12A	−13,517
ADG26-60	MLMM	6	75282198	CNCB10004604	0	1	1.87E-05	A/G	−47.31	EPHA2	2,884
ADG26-60	MLMM	11	100808	CNC10110003	0	1	1.57E-05	A/G	−38.23	ATP12A	−13,517
ADG26-90	BLINK	5	106863651	CNC10052030	0	1	4.27E-06	A/G	−41.01	ZDHHC17	2,469,391
ADG26-90	BLINK	9	13348686	CNC10090293	0	1	1.20E-06	A/G	−73.68	TENM4	0
ADG26-90	GLM	5	106863651	CNC10052030	0	1	1.28E-05	A/G	−56.29	ZDHHC17	2,469,391
ADG26-90	GLM	9	13348686	CNC10090293	0	1	1.04E-05	A/G	−99.84	TENM4	0

1) The number of SNPs that reached the genome significant threshold (p<0.05/50739).

2) The number of SNPs that reached the suggest significant threshold (p<1/50739).

3) The nearest annotated genes from the most significant SNPs.

4) The distance from the most significant SNPs to the nearest genes. Minus indicates SNP locates on the upstream of target gene, positive indicates SNP locates on the downstream of target gene, zero indicates SNP locates in the gene.

BW, body weight; ADG, average daily gain; BLINK, Bayesian-information and LD iteratively nested keyway; GLM, general linear model; MLMM, multiple-locus mixed-model.

## SUPPLEMENTARY MATERIAL

**Supplementary 1**. Descriptive statistics of phenotypes.

**Supplementary 2**. Genome-wide association study (GWAS) analysis of ADG traits.
