# Supplementary material for: Single- and multiple-locus model genome-wide association study for growth traits in Dongliao black pigs
Source: Anim Biosci. 2025 Jul 11;38(11):2312–23. doi: 10.5713/ab.25.0126 (PMC12580961; doi:10.5713/ab.25.0126)
Supplement: Supplementary file 1 [file ab-25-0126-supplementary-1.pdf]

Supplementary 1: Descriptive statistics of phenotypes

Traits	Description	min	max	mean	SD	CV
BW0	Birth weight, kg	0.73	1.86	1.28	0.23	17.79%
BW26	Body weight on age 26 days, kg	2.19	8.64	5.48	1.19	21.68%
BW60	Body weight on age 60 days, kg	4.40	16.50	9.42	2.48	26.28%
BW90	Body weight on age 90 days, kg	6.83	27.31	14.88	4.91	32.98%
ADG0_26	Average daily gain of 0-26 days, g/day	34.42	286.35	161.58	47.08	29.14%
ADG0_60	Average daily gain of 0-60 days, g/day	50.67	263.83	136.01	41.71	30.66%
ADG0_90	Average daily gain of 0-90 days, g/day	65.28	321.83	153.17	57.13	37.30%
ADG26_60	Average daily gain of 26-60 days, g/day	0.88	304.41	124.98	72.50	58.01%
ADG26_90	Average daily gain of 26-90 days, g/day	3.92	393.44	150.84	79.35	52.61%
ADG60_90	Average daily gain of 60-90 days, g/day	8.33	486.83	186.19	94.96	51.00%
