# Supplementary figures and images for: Single- and multiple-locus model genome-wide association study for growth traits in Dongliao black pigs
Source: Anim Biosci. 2025 Jul 11;38(11):2312–23. doi: 10.5713/ab.25.0126 (PMC12580961; doi:10.5713/ab.25.0126)

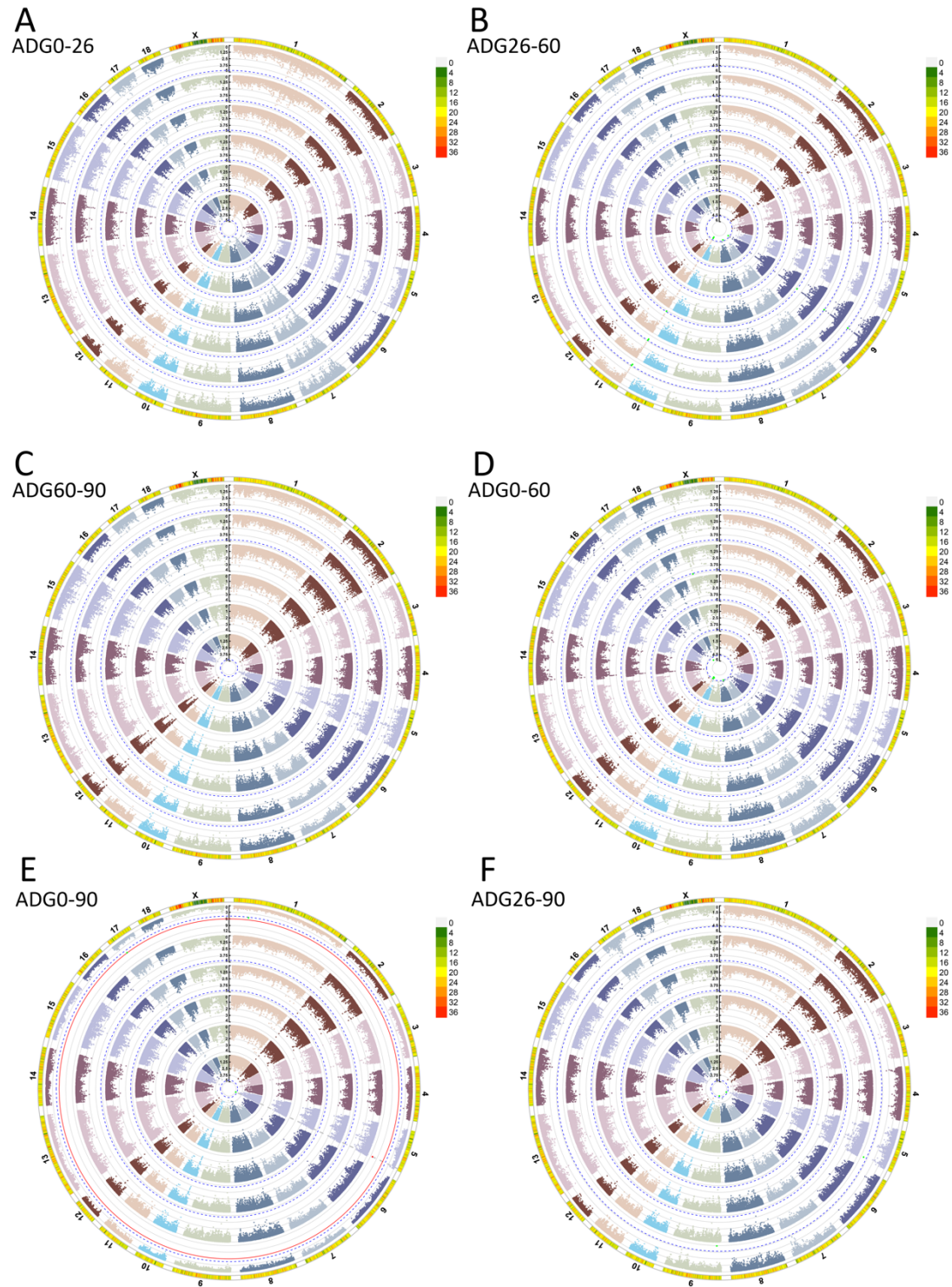

Supplementary 2: Genome-wide association study (GWAS) analysis of ADG traits.

Supplement: Supplementary file 2 [file ab-25-0126-supplementary-2.pdf]
